# Cholelithiasis With a Rare Branched Accessory Hepatic Duct: A Case Report

**DOI:** 10.7759/cureus.82409

**Published:** 2025-04-16

**Authors:** Koki Kawakami, Koji Yasuda, Ryoji Hyakudomi, Jun Otani

**Affiliations:** 1 Surgical Gastroenterology, Unnan City Hospital, Unnan City, JPN

**Keywords:** accessory hepatic duct, bile leakage, cholecystectomy, cholelithiasis, critical view of safety

## Abstract

The patient was a 71-year-old male who underwent laparoscopic cholecystectomy. Preoperative ERCP (Endoscopic Retrograde Cholangiopancreatography) and MRCP (Magnetic Resonance Cholangiopancreatography) did not reveal any obvious biliary tract anomalies. However, during surgery, a vascular structure was observed while dissecting the dorsal side of the gallbladder. The blade of the ultrasonic coagulation and cutting device came into contact with this structure, resulting in bile leakage.

The posterior sectoral hepatic duct was found to merge with the common bile duct dorsally, distal to the confluence with the cystic duct. The damaged site was sutured and closed with 4-0 non-absorbable sutures.

An accessory hepatic duct is a biliary tract anomaly in which an intrahepatic bile duct supplying a subsegmental liver region courses extrahepatically and drains into the common hepatic duct, common bile duct, gallbladder, or cystic duct.

It is crucial to thoroughly evaluate the biliary anatomy preoperatively. Additionally, the surgeon must be well-versed in repair techniques and biliary reconstruction procedures.

## Introduction

The most common complication of laparoscopic cholecystectomy is intraoperative bile duct injury. Although the incidence of intraoperative bile duct injury has been decreasing, it is still reported to occur in 0.66% of cases [1]. In patients with biliary tract anomalies, the frequency of bile duct injury is reported to be 3.2 to 8.4 times higher than in patients with normal anatomy [2-4]. Accessory hepatic ducts represent one form of biliary tract anomaly.

Here, we report a rare case of gallbladder stones with an unusual branching pattern that was not recognized preoperatively, along with a review of the literature.

## Case presentation

The patient was a 71-year-old male. He underwent endoscopic treatment for common bile duct stones in the Department of Gastroenterology at our hospital. He was referred to our department for surgical treatment of gallbladder stones. His past medical history included type 2 diabetes mellitus. He was 164 cm tall, weighed 56 kg, and had a BMI of 20.8 kg/m^2^. Physical examination revealed a flat and soft abdomen with no tenderness.

**Figure 1:**
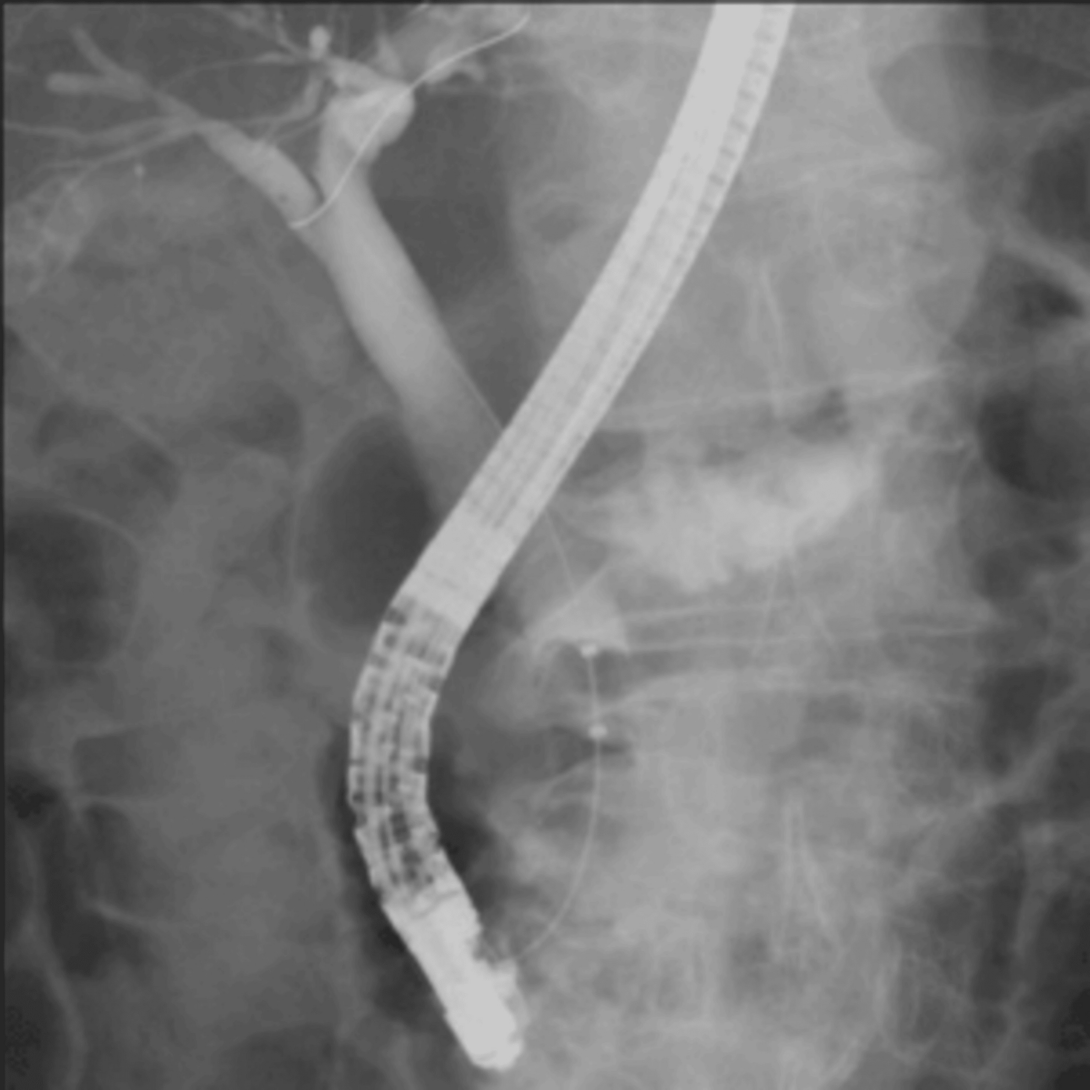
ERCP (Endoscopic Retrograde Cholangiopancreatography)

**Figure 2:**
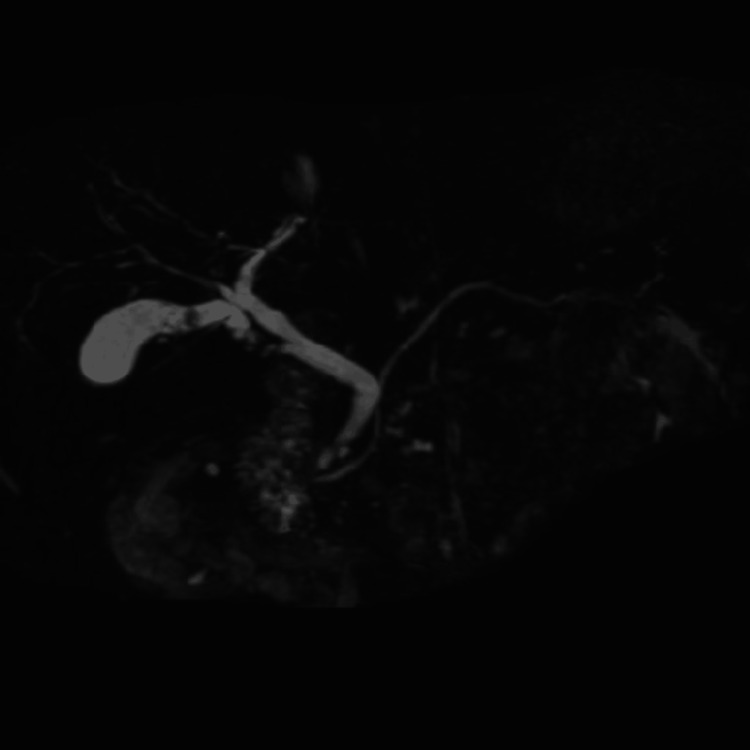
MRCP (Magnetic Resonance Cholangiopancreatography)

Preoperative ERCP (Endoscopic Retrograde Cholangiopancreatography) and MRCP (Magnetic Resonance Cholangiopancreatography) showed that the cystic duct branched off from the common bile duct. No obvious biliary tract anomalies, such as accessory hepatic ducts, were identified preoperatively (Figures 1, 2).

**Figure 3:**
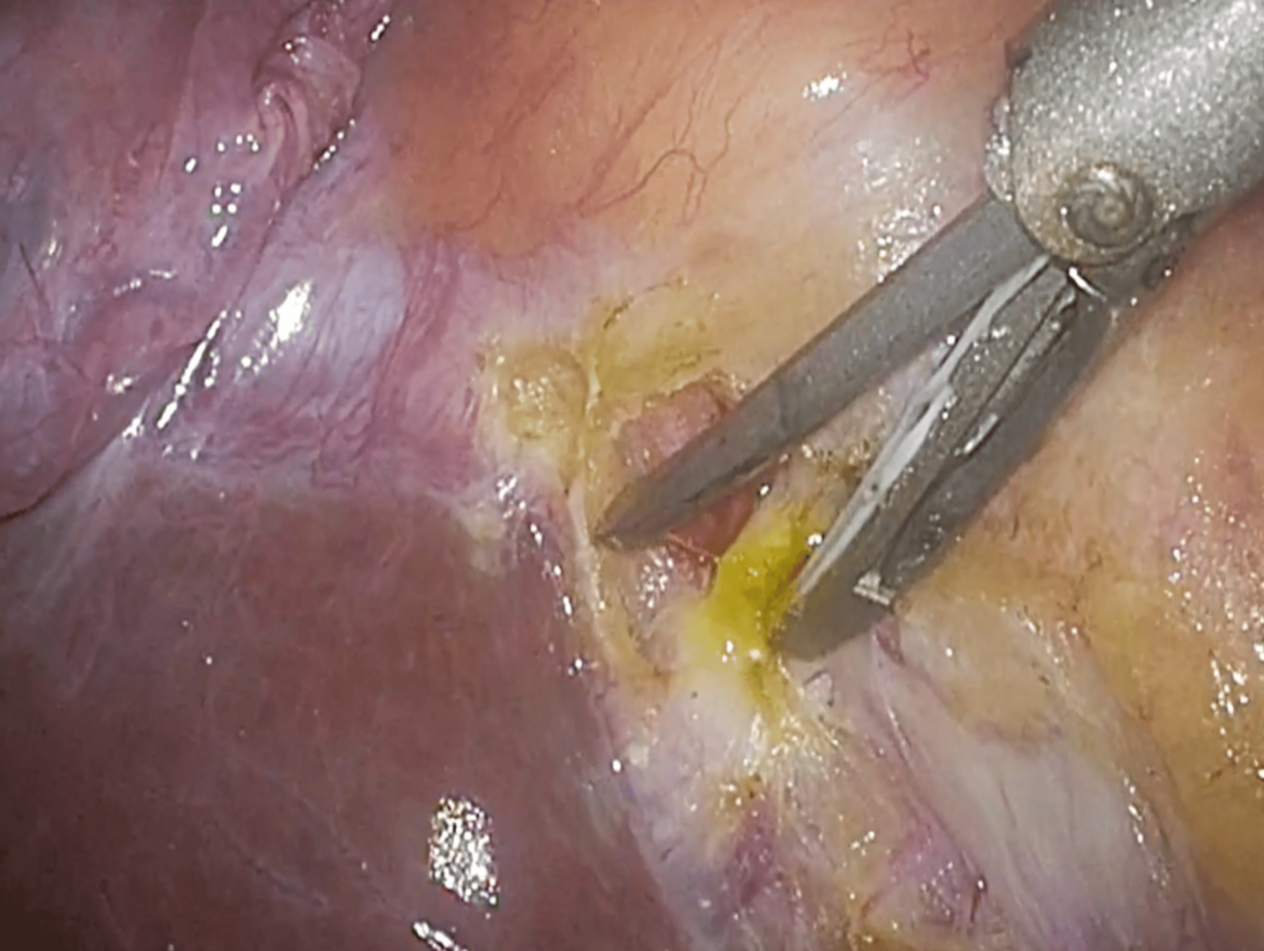
Bile Leakage During dissection of the dorsal side of the gallbladder, a bile duct was inadvertently transected when the blade of the ultrasonic coagulation and cutting device came into contact with it, causing bile leakage.

Intraoperative findings revealed mild inflammation and wall thickening of the gallbladder. The Rouviere's sulcus was identified, and the serosa was incised on its ventral and dorsal sides, exposing the subserosal layer. During dissection of the dorsal side of the gallbladder, a bile duct was inadvertently transected when the blade of the ultrasonic coagulation and cutting device came into contact with it, causing bile leakage (Figure 3).

**Figure 4:**
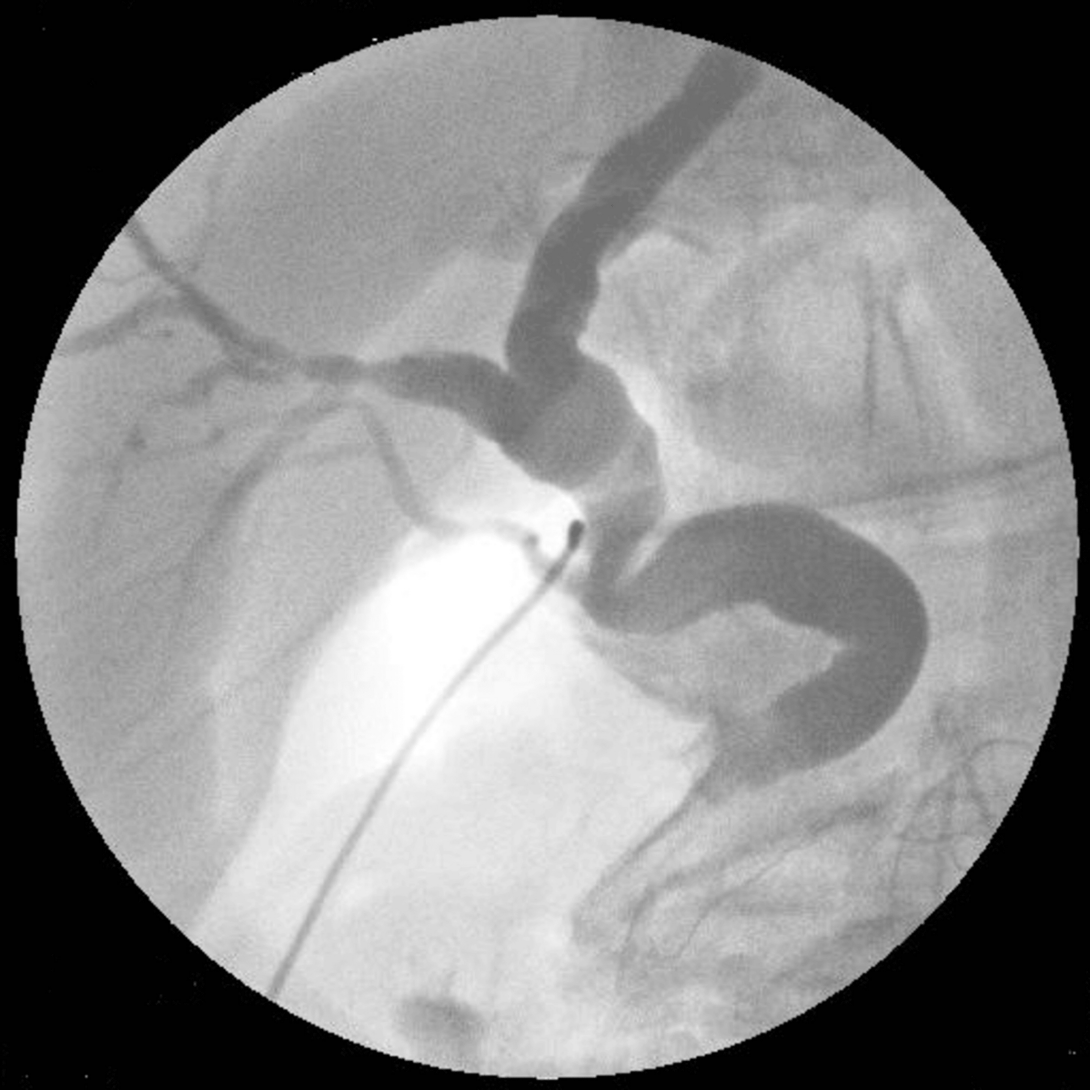
Cholangiography After gallbladder removal, a cholangiography tube was inserted through the cystic duct, revealing that the posterior segmental hepatic duct joined the dorsal side of the common bile duct distal to the cystic duct junction, suggesting injury to the posterior segmental hepatic duct.

After gallbladder removal, a cholangiography tube was inserted through the cystic duct, revealing that the posterior segmental hepatic duct joined the dorsal side of the common bile duct distal to the cystic duct junction, suggesting injury to the posterior segmental hepatic duct (Figure 4).

The injured site was sutured and closed with 4-0 non-absorbable sutures, and the operation was completed.

The postoperative course was uneventful, with no complications such as bile leakage, and the patient was discharged on postoperative day 8. The operative time was 182 minutes, and blood loss was 50 ml.

## Discussion

Accessory hepatic ducts are defined as intrahepatic bile ducts that supply liver regions at the segmental level or below but travel extrahepatically and merge with the common hepatic duct, common bile duct, gallbladder, or cystic duct [5]. Accessory hepatic ducts are reported to occur in 1% of cases, though autopsy studies suggest a prevalence as high as 35%, indicating that they are not rare [6-7].

Patients with biliary tract anomalies have a 3.2 to 8.4 times higher risk of bile duct injury compared to those with normal anatomy, necessitating caution during surgery [2-4]. Reports indicate that in cases where accessory hepatic ducts are not recognized preoperatively, bile duct injury occurs in 50% of patients, a notably high rate.

The diameter of an accessory hepatic duct is proportional to the area it drains [8]. Similar to our case, most reports describe aberrant ducts in the posterior sector; however, there are also reports of aberrant ducts in the anterior sector, left hepatic duct, and caudate lobe branches [9-12].

In Japan, the Hisatsugu classification is commonly used to categorize accessory hepatic ducts. The most frequent anomaly is Type III (57.4%), in which the posterior sectoral branch joins the common hepatic duct. The second most common is Type I (16.8%), where the posterior sectoral hepatic duct joins the common bile duct along with the cystic duct. In our case, the posterior sectoral branch merged with the common bile duct distal to the cystic duct insertion (Type IV, 3.0%), a very rare branching pattern. To prevent unexpected bile duct injury, preoperative imaging such as MRCP and DIC-CT is essential to understand the biliary anatomy.

Laparoscopic surgery, compared to open surgery, offers advantages such as magnification and an upward view from a caudal perspective, allowing for precise exposure of the subserosal inner layer [5]. Due to these factors, laparoscopic surgery is presumed to have a lower risk of bile duct injury in cases with minimal inflammation than open surgery. Even when preoperative recognition of accessory hepatic ducts is lacking, ensuring a Critical View of Safety (CVS) during laparoscopic surgery can prevent most bile duct injuries [5].

However, in Type I and Type V anomalies, there is a risk of misidentifying the cystic duct and cystic artery as accessory hepatic ducts, leading to bile duct injury even with the CVS secured. Furthermore, as seen in our case, an unrecognized accessory hepatic duct may be inadvertently injured during surgery. In cases of unexpected bile duct injury, conversion to open surgery should be considered based on the surgeon’s skill level.

Regarding the management of injured accessory hepatic ducts, it has been reported that ducts with diameters of ≤1-2 mm can be ligated, whereas those ≥2 mm with bile leakage require biliary reconstruction [13-14]. Careful evaluation of each case is necessary to determine the appropriate approach.

## Conclusions

Finally, cholecystectomy is one of the most commonly performed operations by young surgeons. Given the presence of a certain number of biliary tract anomalies, caution is required. Routine preoperative assessment of biliary anatomy and meticulous adherence to the principles of the CVS are crucial. In cases of unexpected bile duct injury, surgeons must be well-versed in biliary reconstruction techniques, selecting appropriate procedures based on the location and extent of the injury.
